# Aptamer-Equipped Protamine Nanomedicine for Precision Lymphoma Therapy

**DOI:** 10.3390/cancers12040780

**Published:** 2020-03-25

**Authors:** Zihua Zeng, Ching-Hsuan Tung, Youli Zu

**Affiliations:** 1 Department of Pathology and Genomic Medicine, Houston Methodist Hospital, Cancer Pathology Research Laboratory, Houston Methodist Research Institute, Houston TX 77030, USA; zzeng@HoustonMethodist.org; 2 Molecular Imaging Innovations Institute, Department of Radiology, Weill Cornell Medical College, New York, NY 10021, USA; cht2018@med.cornell.edu

**Keywords:** aptamer, lymphoma, precision therapy, protamine nanomedicine, targeting delivery

## Abstract

Anaplastic large cell lymphoma (ALCL) is the most common T-cell lymphoma in children. ALCL cells characteristically express surface CD30 molecules and carry the pathogenic ALK oncogene, both of which are diagnostic biomarkers and are also potential therapeutic targets. For precision therapy, we report herein a protamine nanomedicine incorporated with oligonucleotide aptamers to selectively target lymphoma cells, a dsDNA/drug payload to efficiently kill targeted cells, and an siRNA to specifically silence ALK oncogenes. The aptamer-equipped protamine nanomedicine was simply fabricated through a non-covalent charge-force reaction. The products had uniform structure morphology under an electron microscope and a peak diameter of 103 nm by dynamic light scattering measurement. Additionally, flow cytometry analysis demonstrated that under CD30 aptamer guidance, the protamine nanomedicine specifically bound to lymphoma cells, but did not react to off-target cells in control experiments. Moreover, specific cell targeting and intracellular delivery of the nanomedicine were also validated by electron and confocal microscopy. Finally, functional studies demonstrated that, through combined cell-selective chemotherapy using a drug payload and oncogene-specific gene therapy using an siRNA, the protamine nanomedicine effectively killed lymphoma cells with little toxicity to off-target cells, indicating its potential for precision therapy.

## 1. Introduction

Lymphomas are cancers originating from the lymphatic system. Based on clinical presentation and therapeutic approach, lymphomas are divided into either Hodgkin lymphoma or non-Hodgkin lymphoma, which includes B-cell and T-cell lymphomas [1]. Anaplastic large cell lymphoma (ALCL) is the most common T-cell lymphoma in children and the second most common aggressive systemic T-cell lymphoma in adults [2,3,4]. Molecular studies demonstrated that the majority of ALCL cases have abnormal expression of the anaplastic lymphoma kinase (ALK) oncogene due to chromosomal translocation t(2;5)(p23;q35), which is a key pathogenic factor for ALCL development. In addition, ALCL cells have a high level of aberrant expression of the CD30 protein, which is a diagnostic biomarker and therapeutic target specific for ALCL. Currently, a multidrug chemotherapy regimen is the standard treatment for ALCL, as well as other aggressive T-cell lymphomas [5,6,7,8]. However, chemotherapy is not cell- or gene-specific, and as a result, it may induce serious side effects that adversely affect patients [9,10,11]. To avoid side effects, tumor-specific therapeutic approaches are required.

For specific lymphoma therapy, the CD30-specific antibody–drug conjugate brentuximab vedotin has been approved by the FDA to treat relapsed/refractory ALCL [12,13]. In addition, gene therapy approaches, including RNA interference therapeutics to silence the ALK oncogene in ALCL tumor cells, have also been investigated [14,15]. However, treatments with a single therapeutic modality may induce drug resistance of lymphoma cells and may not be potent enough to fully eradicate the disease. Therefore, the development of new targeted therapeutics that can treat ALCL more effectively through a combination of multiple therapeutic modalities is required.

Nanotechnology is an attractive delivery platform that can carry multiple therapeutic modalities for cancer treatments. Non-viral nanoparticle delivery systems, including cationic polymers, metal or magnetic particles, lipid and liposome molecules, and protein complexes, have been widely investigated [16,17,18,19]. Due to different formulation components, nanoparticles vary in physical properties, chemical features, and biological activities. For clinical applications, biocompatible and biodegradable nanoparticle systems are required. Protamine is an arginine-rich peptide with a molecular mass of approximately 5 kDa. Importantly, sulfate salt forms of protamine are considered to be biologically safe, and have been adopted for clinical use as an FDA-approved antidote for heparin overdose, as a stabilizer in vaccines, and for the sustained release of insulin in treating diabetes [20,21,22,23]. Therefore, protamine can serve as a risk-free nanomedicine carrier with multiple therapeutic modalities that achieve high therapeutic efficacy.

For precision cancer therapy, specific delivery of therapeutics to target tumor cells is critical to enhance therapy efficacy and, more importantly, to reduce adverse side effects in normal tissues and organs. To this end, nanoparticles have been conjugated with different ligand molecules, including antibodies, transferrin, RGD, folate, cholesterol, and aptamers [24]. Aptamers are a class of small molecular ligands composed of short, single-stranded oligonucleotides (RNA or ssDNA) ranging from 30 to 70 bases in length [25,26,27]. Oligonucleotide aptamers can specifically recognize a range of targets, including small molecules, macromolecules, viruses, live cells, and tissues [28]. Functionally similar to protein antibodies, aptamers bind to their cognate targets with high affinity because of their unique three-dimensional structure. Notably, aptamers offer several advantages for targeted delivery in vivo because they possess faster cell-binding abilities and quicker tissue penetration capacity due to their smaller size [29,30,31,32].

We report herein a CD30 aptamer-equipped protamine nanomedicine made entirely of biocompatible materials and capable of carrying both chemotherapeutic drugs and an ALK oncogene-specific siRNA. Therefore, under aptamer guidance, this protamine nanomedicine is able to specifically target lymphoma cells, and thus deliver cell-selective chemotherapy and oncogene-specific gene therapy. These findings provide a new avenue for developing biocompatible nanomedicine for precision lymphoma therapy.

## 2. Results

### 2.1. Fabrication and Characterization of Protamine Nanoparticles

**Figure 1:**
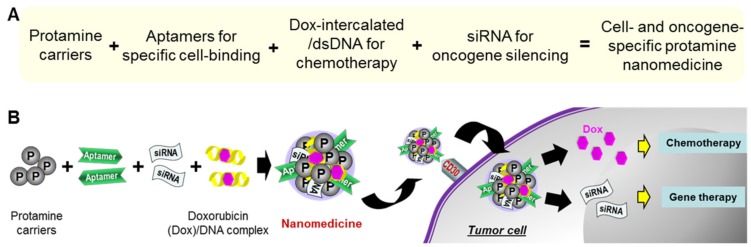
(**A**) Fabrication scheme of protamine nanomedicine. (**B**) Precision cancer therapy by protamine nanomedicine.

To investigate precision ALCL treatment, we utilized the facts that lymphoma cells aberrantly express high levels of surface CD30 and have an active pathogenic ALK oncogene due to abnormal chromosomal translocation. We thus used RNA-based CD30-specific aptamers for selective cell targeting [33,34,35], an ALK oncogene-specific siRNA for gene therapy [36,37], and the chemotherapeutic drug doxorubicin (Dox) in a DNA intercalation form to kill targeted lymphoma cells, as described in the Materials and Methods Section. To assemble these functional agents, protamine was employed as a carrier [38,39]. The cell- and oncogene-specific protamine nanomedicine was fabricated by simply incorporating negatively charged nucleotides, including aptamers, siRNAs, and dsDNA/Dox complex, with a positively charged protamine through a non-covalent reaction (Figure 1A). We expected that, under aptamer guidance, the nanomedicine would selectively target lymphoma cells and mediate intracellular delivery of the siRNA and Dox payload for specific oncogene silencing and cell-selective chemotherapy, as illustrated in Figure 1B.

**Figure 2:**
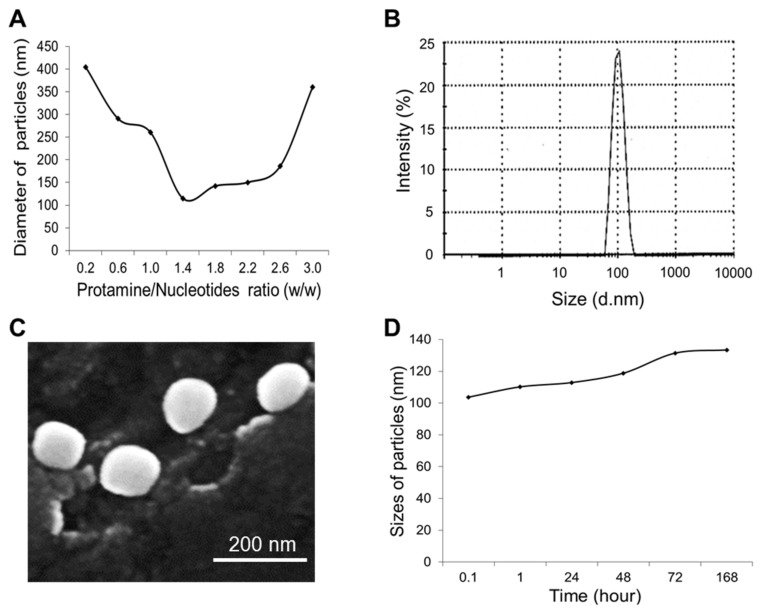
Characterization of protamine nanoparticles. (**A**) Fabrication of nanoparticles at different ratios of protamine to nucleotides, weight/weight (*w*/*w*). The most compact nanoparticles were fabricated at a 1.5:1 ratio of protamine to nucleotides. (**B**) Dynamic light scattering measurement (DLS) of the protamine nanoparticles detected a peak size of 103 nm. (**C**) Scanning electronic microscopy revealed uniform protamine nanoparticles with the size consistent with DLS measurement. (**D**) Stability study of protamine nanoparticles by DLS measurement to kinetically monitor size changes up to seven days post-production, ranging from 103 nm to 133 nm. The data shown represent three independent experiments with similar results.

To optimize the fabrication conditions of the nanomedicine, test protamine nanoparticles were initially produced. First, the nucleotide mixtures were made of equal amounts of RNA aptamers and dsDNA, weight/weight (*w*/*w*). Next, protamine was added into the nucleotide mixtures at a ratio of 0.2/3.0 (*w*/*w*) to produce the test nanoparticles. The sizes of the fabricated protamine nanoparticles were then detected by dynamic light scattering (DLS) measurement. Figure 2A shows that the most compact protamine nanoparticles were generated at a 1.5/1.0 ratio (*w*/*w*) of protamine to nucleotide mixtures, with a peak size of 103 nm in diameter (Figure 2B). In addition, the test nanoparticles were also examined by scanning electronic microscopy and showed uniform structure and size, consistent with DLS measurement (Figure 2C). For stability studies, nanoparticles were stored in phosphate-buffered saline (PBS) at 4 °C for up to seven days, and changes in size were kinetically monitored by DLS measurement. Figure 2D shows that the protamine nanoparticles were stable and had minimal size changes from 103 to 133 nm in diameter at seven days post-production. Therefore, in the following studies, nanoparticles and nanomedicine were fabricated at a 1.5/1.0 ratio of protamine to total nucleotide components.

**Figure 3:**
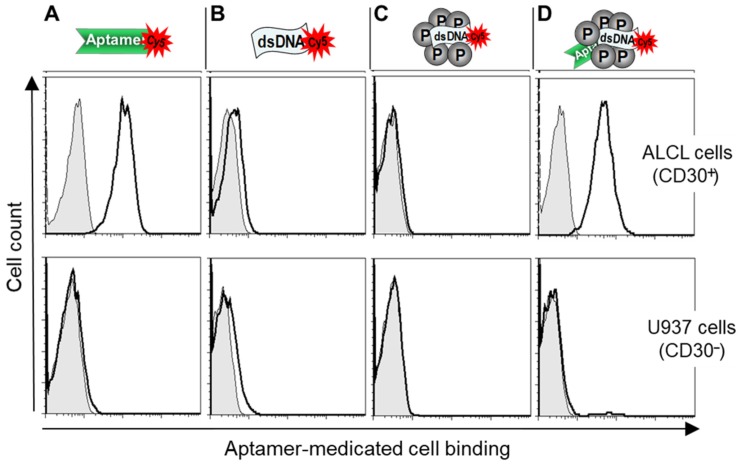
Aptamer-guided specific cell binding of the protamine nanoparticles. Cultured lymphoma cells, CD30-expressing Karpas 299 cells (upper panel), and CD30-negative U937 cells (lower panel), were treated with (**A**) an RNA-based CD30 aptamer probe alone, (**B**) synthetic dsDNA alone, (**C**) protamine nanoparticles composed of dsDNA, or (**D**) protamine nanoparticles composed of both CD30 aptamer and dsDNA. Resultant cell binding was evaluated by flow cytometry analysis. The data shown represent three independent experiments with similar results.

To study the cell-binding capacity, the test nanoparticles were formulated with Cy5-labeled dsDNAs and incubated with cultured lymphoma cells. Flow cytometry analysis showed that the protamine nanoparticles specifically bound to ALCL lymphoma cells (Karpas 299), but did not react to CD30-negative control cells (U937) (Figure 3). Notably, the observed cell-binding pattern was nearly identical to that of the CD30-specific aptamer probe alone. In contrast, no cell binding was detected in control experiments with dsDNA alone or nanoparticles that lacked aptamer sequences under the same conditions. These findings confirmed the aptamer-guided specific cell binding of the test nanoparticles.

### 2.2. Functional Analysis of Protamine Nanoparticles

**Figure 4:**
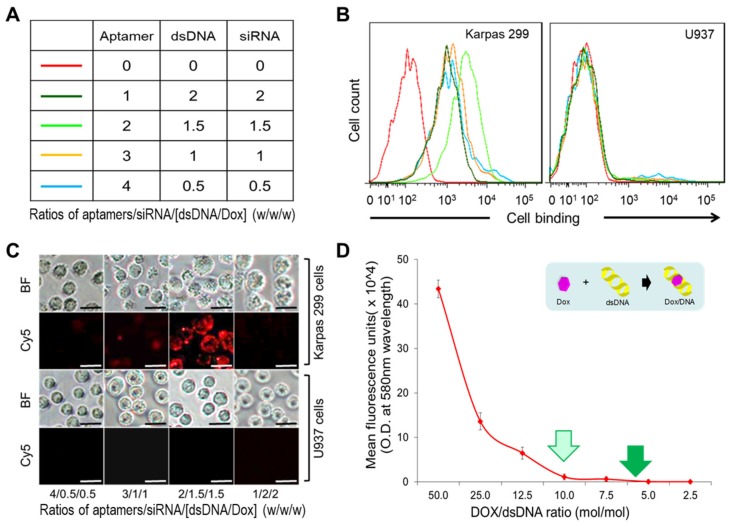
Optimizing protamine nanoparticle products. (**A**) To optimize cell-targeting capacity, testing nanoparticles were fabricated with a 1.5:1.0 ratio of protamine to total nucleotide components, but different ratios of aptamers, dsDNA, and siRNA (*w*/*w*/*w*), as listed. (**B**) Cell binding of the protamine nanoparticles was examined by flow cytometry analysis. The highest cell binding to Karpas 299 cells was seen at a 2.0/1.5/1.5 ratio of aptamers, dsDNA, and siRNA components, with no reaction to control U937 cells. (**C**) Cell-binding capacities of individual protamine nanoparticles were also confirmed by fluorescent microscopy with similar results. (**D**) To characterize Dox drug loading into synthetic dsDNA sequences, the dsDNA/Dox complex formation was monitored by detecting fluorescent signals of free Dox leftover in reactions using a microplate reader. A drug-loading rate greater than 98% was achieved at a 10:1 ratio of Dox to dsDNA (mol/mol), and nearly complete loading of Dox into dsDNA was seen in reactions with a 6:1 ratio of Dox to dsDNA. The data shown in B and C represent three independent experiments with similar results. The data shown in D represent mean ± SD, *n* = 3 independent experiments. (Scale bar = 20 µm).

To obtain maximal cell targeting capacity, a series of protamine nanoparticles were fabricated with defined amounts of total nucleotide components, but different ratios of aptamers/dsDNA/siRNA (*w*/*w*/*w*), as indicated in Figure 4A. For cell-binding studies, protamine nanoparticles were generated using Cy5-labeled dsDNAs for tracking purposes. Cultured lymphoma cells were treated with the nanoparticles and resultant cell binding was quantified by flow cytometry analysis. Figure 4B shows that the protamine nanoparticles with aptamers/dsDNA/siRNA at a ratio of 2.0/1.5/1.5 had the highest targeting affinity to Karpas 299 cells, but did not react to control U937 cells under the same treatment conditions. In addition, results of cell-binding studies of the protamine nanoparticles were also confirmed by fluorescent microscopy (Figure 4C). Therefore, the protamine nanomedicine was fabricated using this formula of nucleotide components with a 2.0/1.5/1.5 ratio of aptamers/siRNA/[dsDNA/Dox] (*w*/*w*/*w*), for the following studies.

### 2.3. Specific Cell Binding and Selective Intracellular Delivery of the Protamine Nanomedicine

For drug loading, Dox was incubated with synthetic dsDNA sequences at different ratios, as indicated in Figure 4D. Dox/dsDNA complex formation was monitored by detecting free Dox left in reactions using a microplate reader because free Dox is fluorescent and its DNA-intercalated form is optically silent. Drug-loading assays revealed that a greater than 98% drug-loading rate was achieved at a 10:1 ratio of Dox to dsDNA (mol/mol), and no free Dox was detected in reactions with a 6:1 ratio of Dox to dsDNA. Therefore, the latter condition was used to produce the nanomedicine in the following studies.

**Figure 5:**
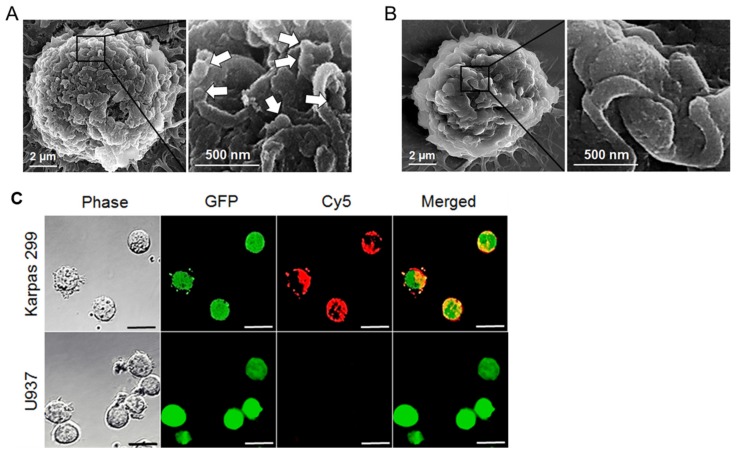
Functional study of protamine nanomedicine. (**A**) Cell-targeting study by scanning electron microscopy (SEM). Specific binding of the protamine nanomedicine to Karpas 299 cells is indicated with arrows. (**B**) In contrast, no protamine nanomedicine was detected on control U937 cells. (**C**) To study intracellular delivery, eGFP-expressing cells (green fluorescence) were treated with Cy5-labeled protamine nanomedicine (red fluorescence) and then examined under the confocal microscope (scale bar = 20 µm). The merged fluorescent image revealed specific binding of the nanomedicine to Karpas 299 cells (red signal on the cell surface) and also confirmed intracellular delivery of the protamine nanomedicine by the presence of intracellular yellow fluorescent signal, which was derived from merged green and red fluorescent signals. The images shown represent three independent experiments with similar results.

To validate cell-binding specificity, cultured cells were treated with the protamine nanomedicine and examined by scanning electron microscopy. Figure 5A,B show that the nanomedicine selectively bound to Karpas 299 cells, but did not react to control U937 cells, demonstrating aptamer-guided specific cell targeting.

We expected that aptamer-mediated cell binding would trigger internalization and intracellular delivery of the nanomedicine, as illustrated in Figure 1B. To confirm this hypothesis, cultured lymphoma cells that stably expressed green fluorescent protein (GFP) were treated with Cy5-labeled nanomedicine, and then examined under a confocal fluorescent microscope. The merged images showed specific cell binding of the nanomedicine to Karpas 299 cells (the red fluorescent signal on the cell surface). More importantly, intracellular delivery of the nanomedicine was also observed in Karpas 299 cells by detecting the yellow fluorescent signal (Figure 5C), which represented the merged signals of cellular GFP (green) and the protamine nanomedicine containing Cy5-labeled dsDNAs (red). In contrast, no nanomedicine signals were detected on or in off-target U937 cells under the same experimental conditions.

### 2.4. Cell-Selective and Oncogene-Specific Effects of the Protamine Nanomedicine on Lymphoma Cells

To validate the therapeutic potential of individual functional nucleotides, a series of protamine nanoparticles were fabricated with different nucleotide components as listed in Table 1.

**Figure 6:**
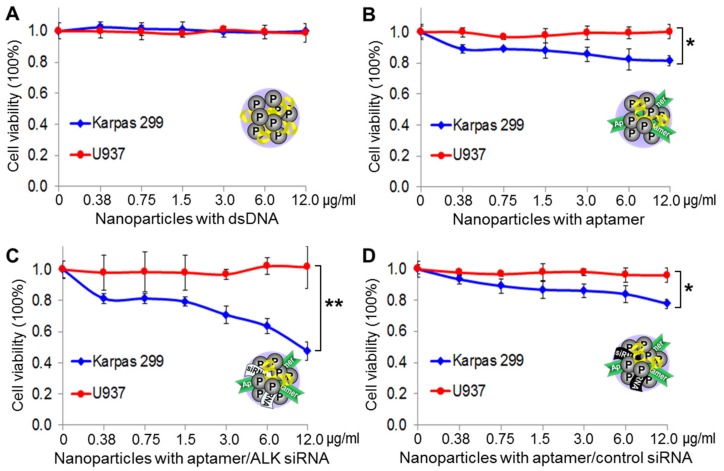
Cellular effects of protamine nanoparticles. Lymphoma cells were exposed to a series of protamine nanoparticles, as indicated. Cell viability was then evaluated by counting viable cells using the trypan blue exclusion method three days post-treatment. (**A**) Protamine nanoparticles composed of dsDNA alone had no effect on the viability of Karpas 299 or U937 cells. (**B**) Protamine nanoparticles composed of CD30 aptamers caused 19% decrease in viability of Karpas 299 cells, but no effect on U937 cells. (**C**) Protamine nanoparticles composed of the aptamer and siRNA specific for ALK oncogene caused death in 53% of Karpas 299 cells, but had no effect on off-target U937 cells. (**D**) In contrast, protamine nanoparticles composed of aptamers and a non-relevant control siRNA had no additional effect on Karpas 299 cells, compared to (B). The data shown represent mean ± SD, *n* = 3 independent experiments; * *p* < 0.05, ** *p* < 0.01.

Cultured lymphoma cells were exposed to the same amounts of nanoparticle products at 37 °C for one hour. The treated cells were then washed to remove free nanoparticles and continually cultured in fresh medium for three days. Resultant changes in cell viability were evaluated with the trypan blue stain. Figure 6A shows that protamine nanoparticles composed of dsDNA alone had no effect on cell viability because they lacked both targeting and therapeutic agents. In contrast, the protamine nanoparticles composed of aptamers alone had a minor effect on Karpas 299 cells with up to 16% decreased cell viability, probably due to an aptamer-mediated cellular effect because it was not seen in off-target U937 cells (Figure 6B). Interestingly, treatment of protamine nanoparticles composed of both aptamers and the ALK oncogene-specific siRNA induced significant death (up to 50%) of Karpas 299 cells, but had no adverse effects on off-target U937 cells (Figure 6C). Notably, no additional cellular effects were seen with nanoparticles composed of aptamers and a non-relevant control siRNA (Figure 6D).

**Figure 7:**
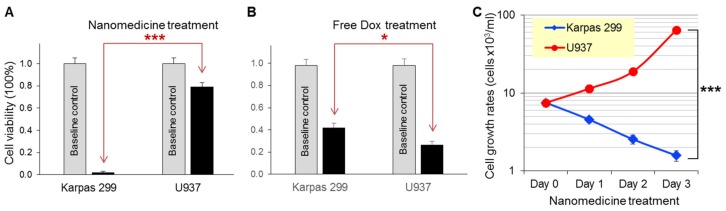
Precision therapeutic effects of protamine nanomedicine. (**A**) Lymphoma cells were treated with the protamine nanomedicine (1 µg/mL) or vehicle alone for baseline control for three days. Cell viability assays showed a 98.5% death rate of Karpas 299 cells versus a 21% death rate of U937 cells under the same treatment conditions. (**B**) In contrast, treatments with the same amount of free Dox drug (1.2 µM) non-discriminatingly killed both Karpas 299 and U937 cells, with more toxicity to U937 cells. (**C**) Cell growth analysis demonstrated the precision cancer therapy potential of the protamine nanomedicine, effectively killing Karpas 299 cells and having no significant effect on the growth of off-target U937 cells under the same treatment conditions. The data shown represent mean ± SD, *n* = 3 independent experiments; * *p* < 0.05, *** *p* < 0.001.

For in vitro therapeutic studies, the protamine nanomedicine was fabricated as described above, which composed of aptamers/siRNA/[dsDNA/Dox] at a 2.0/1.5/1.5 ratio (*w*/*w*/*w*). Cultured cells were exposed to the nanomedicine (1 µg/mL) at 37 °C for one hour. The treated cells were then washed to remove free nanomedicine and continually cultured in fresh medium for three days. Resultant changes in cell viability were detected by the trypan blue exclusion method. Figure 7A shows that nanomedicine treatment killed nearly all Karpas 299 cells, with a 98.5% death rate likely caused by the combined effects of aptamer-guided cell targeting, siRNA-mediated oncogene silencing, and Dox payload-induced chemotherapy. In contrast, under the same treatment conditions, the nanomedicine showed mild toxicity to off-target U937 cells with a 21% death rate, probably due to the non-cell-specific effect of free Dox drug. Moreover, when compared to the protamine nanomedicine, targeted chemotherapy alone by aptamer nanoparticles carrying Dox/dsDNA had less effects on Karpas 299 cells under the same treatment conditions (Figure S1).

To rule out bias of drug sensitivity, lymphoma cells were exposed to the same amounts of free Dox (1.2 µM) under the same treatment conditions in control experiments. Figure 7B shows that free Dox treatment non-discriminatingly killed Karpas 299 and U937 cells with 58% and 73% death rates, respectively, thus implying that the control U937 cells were more sensitive to free Dox treatment. These findings demonstrated that the nanomedicine was cell-specific, and thus had high therapeutic efficacy in targeted cells and low toxicity to off-target cells.

Finally, to compare cellular effects, both Karpas 299 and U937 cells were treated with the nanomedicine (1 µg/mL) under the same condition as described above. Cell growth rates were kinetically monitored for three days by cell-counting assays with trypan blue staining. Figure 7C shows that the nanomedicine had the capacity to not only efficiently kill Karpas 299 cells with a 4.6-fold decrease in cell growth, but also had no significant toxicity to off-target U937 cells, which had 8.5-fold increased growth.

## 3. Discussion

For precision cancer treatment, therapeutics need to be tumor cell-selective and/or oncogene product-specific to achieve high therapeutic efficacy on cancer cells and no toxicity to off-target normal tissues. In addition, a logical combination of multiple therapeutic modalities is a powerful approach to avoid the development of drug resistance by tumor cells. To this end, we developed an aptamer-equipped protamine nanomedicine. In vitro studies showed that under aptamer guidance, the protamine nanomedicine was able to specifically bind lymphoma cells, but did not react to off-target cells (Figure 5). It has been reported that aptamer-mediated cell binding can trigger both endocytosis and macropinocytosis by targeted cells [37]. Endocytosis into enzyme-rich lysosomes will result in the degradation of the protamine nanomedicine and the rapid release of Dox payload to exclusively kill the targeted cells. Simultaneously, macropinocytosis of the protamine nanomedicine by the targeted cells will lead to the intracellular delivery of intact siRNA to silence ALK oncogenes and inhibit lymphoma cell proliferation. Thus, we conclude that our nanomedicine treatment caused significantly higher death rates in lymphoma cells and a much lower effect on off-target cells than treatments with equal amounts of free Dox drug alone (Figure 7A,B). Therefore, through combined chemotherapy and gene therapy, our nanomedicine is ideal for precision therapy (Figure 7C).

Although a variety of nanomaterials have been reported, only a few have been studied for clinical use, largely because of safety concerns regarding their chemical components and the complexity of the fabrication processes. Our protamine nanomedicine is highly biocompatible because it is made of clinically used protamine carriers and biodegradable oligonucleotides that have different functions, including the RNA-based aptamer, dsDNA/Dox complex, and siRNA specific for the ALK oncogene. Notably, the incorporation of RNA-based aptamers and siRNAs into nanomedicine may enhance their stability for targeted delivery. Moreover, fabrication of the protamine nanomedicine is a simple one-step reproducible process and does not involve a covalent chemical reaction, indicating the possibility for large-scale production for clinical use.

Taken together, protamine nanomedicine technology has high translational potential for clinical use in precision lymphoma therapy. Importantly, this technology could be a universal platform for precision therapy to treat different cancers by logical replacement of aptamers for target delivery as well as drug payloads and/or siRNAs for cell-selective and/or gene-specific therapy. For further validation, pre-clinical in vivo studies with animal models are ongoing, and findings will be reported when completed.

## 4. Materials and Methods

### 4.1. Reagents and Cell Lines

Protamine sulfate and doxorubicin hydrochloride were purchased from Sigma-Aldrich (St. Louis, MO, USA). A protamine stock solution was prepared with deionized water (1 mg/mL) and stored at −20 °C until use. A doxorubicin solution was prepared with deionized water immediately before use.

All oligonucleotides were synthesized by IDT (Integrated DNA Technologies, Coralville, IA, USA), including the RNA-based CD30-aptamer:

5’-GAUUCGUAUGGGUGGGAUCGGGAAGGGCUACGAACACCG-3’ (33–35), siRNA specific for ALK oncogene: sense sequence 5’-CACUUAGUAGUGUACCGCCTT-3’ and antisense sequence 5’-GGCGGUACACUACUAAGUGTT-3’ (36,37), and dsDNA: sense sequence 5’- CGCGCGCGCGCGCGCGCGCG-3’ and antisense sequence 5’-CGCGCGCGCGCGCGCGCGCG-3’.

Karpas 299 cells, a CD30-positive cell line of human ALCL, were obtained through collaboration with Dr. Mark Raffeld at NCI/NIH, and U937 cells, a CD30-negative cell line of human histiocytic lymphoma, were purchased from ATCC (American Type Culture Collection, Manassas, VA, USA). Cells were cultured in RPMI 1640 media with 10% FBS, 100 U/mL penicillin, and 100 μg/mL streptomycin at 37 °C in an atmosphere of 5% CO_2_ and 95% humidity.

### 4.2. Fabrication and Characterization of Protamine/Nucleotide Nanoparticles

To fabricate protamine nanoparticles and nanomedicine, 7.5 µg protamine was mixed with 50 µL deionized water in 1.5 mL Eppendorf tubes. Different amounts (37.5 µg to 2.5 µg) of nucleotides (aptamers, dsDNA, ALK-siRNAs, and/or dsDNA/Dox complex) were mixed with 950 µL deionized water in 1.5 mL Eppendorf tubes. The protamine solution was added to the nucleotide mixture and mixed well by vortex briefly to form the nanoparticles and nanomedicine. Resultant products were stored at 4 °C until use.

Sizes of produced protamine nanoparticles were measured by DLS with a Zetasizer Nano S instrument (Malvern Instruments Ltd., Worcestershire, United Kingdom). To optimize production conditions, different ratios of protamine to total nucleotides were tested, ranging from 0.2 to 3.0 (*w*/*w*), and resultant sizes of nanoparticles were evaluated by DLS measurement as indicated. For stability study, changes in nanoparticle size were kinetically monitored by DLS measurement for up to seven days post-fabrication in PBS.

### 4.3. Scanning Electron Microscopy

To confirm the size and shape, fabricated nanoparticles were loaded on silicon slices and fixed with 2.5% glutaraldehyde (Sigma-Aldrich, St. Louis, MO, USA) at 4 °C for two hours, followed by sequential dehydration for 10 min each in 20%, 30%, 50%, 70%, 90%, and 100% ethanol. The fixed nanoparticles were sputter-coated with PtPd using a Cressington 208 HR Sputter Coater (Cressington Scientific, Cranberry Twp, PA, USA) and examined by a scanning electron microscope (Nova NanoSEM 230, FEI Co., Hillsboro, OR, USA).

To confirm specific cell binding, cultured cells were incubated with the protamine nanomedicine at a final concentration of 1.2 µg in 100 µL PBS at room temperature for 30 min. After washing twice with PBS, the treated cells were loaded on silicon slices and then fixed with 2.5% glutaraldehyde at 4 °C for two hours and examined by a scanning electron microscope (Nova NanoSEM 230, FEI Co., Hillsboro, OR, USA).

### 4.4. Cell Binding Assays

To evaluate aptamer-mediated cell binding, cultured cells (2 × 10^5^) were treated with synthetic RNA-based CD30 aptamers alone or dsDNA alone at a final concentration of 50 nM, or freshly-prepared protamine nanoparticles containing the same amounts of aptamer and/or dsDNA in 100 µL PBS. For tracking purposes, nucleotide components were labeled with Cy5, as illustrated. For 20 min post-incubation, cells were washed with PBS to remove free products and resuspended in 400 µL PBS. Resultant cell binding was quantified by flow cytometry analysis with a LSRII flow cytometer (BD Biosciences, San Jose, CA, USA) and FlowJo (v7.0) software. In addition, the same sets of treated cells were also examined under an Olympus IX81 fluorescent microscope (Olympus America, Center Valley, PA, USA) simultaneously to observe cellular fluorescent signals and confirm aptamer-mediated cell binding of the protamine nanoparticles.

To achieve maximal cell binding, the test nanoparticles were fabricated by mixing protamine with equal amounts of nucleotides containing different ratios of aptamer/dsDNA/siRNA (1.0/2.0/2.0, 2.0/1.5/1.5, 3.0/1.0/1.0, and 4.0/0.5/0.5 (*w*/*w*/*w*)). Cultured cells were treated with individual products as described above, and resultant cell binding was quantified by flow cytometry analysis as well as fluorescent microscopy examination.

### 4.5. Intracellular Delivery Assays

Protamine nanomedicine was fabricated using a 1.5:1 ratio of protamine to total nucleotide components (*w*/*w*), which contained aptamer/dsDNA/siRNA at a 2.0/1.5/1.5 (mol/mol) ratio. For tracking purposes, dsDNAs were labeled with a Cy5 fluorescent reporter. To validate intracellular delivery, cultured cells with stable expression of GFP were treated with the nanomedicine at 37 °C for one hour. Cells were then harvested, fixed with 4% formaldehyde for 10 min, washed with PBS, and suspended in PBS. Finally, 15 μL of cell solution was loaded onto glass slides, covered with a coverslip, and examined using a Fluo ViewTM 1000 confocal microscope (Olympus America, Center Valley, PA, USA). The presence of merged fluorescent signals (yellow) derived from the nanomedicine (red) and cytoplasm (green) indicated intracellular delivery.

### 4.6. Loading of Doxorubicin into dsDNAs

Doxorubicin drug (Dox) loading was conducted through a simple self-loading process. Notably, because of its chemical properties, Dox can freely intercalate into dsDNAs via a non-covalent process. To optimize Dox-loading conditions, 100 µL of Dox (10 µM) was mixed with equal volumes of dsDNA with concentrations ranging from 0.2 to 4.0 µM. The formation of the Dox-intercalated dsDNA complex was determined by monitoring free Dox drug leftover in reactions by a microplate reader as free Dox drug is fluorescent and its DNA intercalation form is optically silent.

### 4.7. Cell Function Assays

To determine therapeutic potential, cultured lymphoma cells were exposed to the nanomedicine at 37 °C for one hour. The treated cells were then washed to remove unbound nanomedicine and continually cultured in fresh medium for three days. Cell viability was determined by the trypan blue exclusion method (ThermoFisher Scientific, Waltham, MA, USA). Cells stained blue were considered non-viable and manually counted under a light microscope. Cell viability was calculated as the number of viable cells divided by the total number of cells using the formula: % viable cells = [1.00 – (number of blue-stained cells/number of total cells)] × 100. In addition, cell growth rates were kinetically monitored by counting absolute viable cells in each experiment with the trypan blue exclusion method at different time points as indicated.

## 5. Conclusions

In this study, we investigated an aptamer-equipped protamine nanomedicine to target ALCL lymphoma cells. The protamine nanomedicine was simply fabricated through a programmatic non-covalent charge-force reaction to incorporate functional oligonucleotides and a drug payload. The nanomedicine products had uniform nanostructure and were biostable. Under aptamer guidance, the nanomedicine specifically targeted lymphoma cells and delivered an siRNA and a drug payload exclusively into cells of interest. Functional studies revealed that through combined cell-selective chemotherapy and oncogene-specific gene therapy, the nanomedicine effectively killed lymphoma cells with little toxicity to off-target cells, demonstrating its potential for precision lymphoma therapy.

## Figures and Tables

**Table 1:** Components of protamine nanoparticles.

Figure	Protamine (ng)	Aptamer (ng)	dsDNA (ng)	ALK-siRNA (ng)	Control RNA (ng)
Figure 6A	1200	-	800	-	-
Figure 6B	1200	320	480	-	-
Figure 6C	1200	320	240	240	-
Figure 6D	1200	320	240	-	240

## Supplementary Materials

The following are available online at https://www.mdpi.com/2072-6694/12/4/780/s1, Figure S1: Therapeutic effects of target chemotherapy alone vs. combined chemotherapy and gene therapy.
