# Supplementary material for: Aptamer-Equipped Protamine Nanomedicine for Precision Lymphoma Therapy
Source: Cancers (Basel). 2020 Mar 25;12(4):780. doi: 10.3390/cancers12040780 (PMC7226387; doi:10.3390/cancers12040780)
Supplement: Supplementary file 1 [file cancers-12-00780-s001.pdf]

Aptamer-equipped protamine nanomedicine for precision lymphoma therapy

Zihua Zeng[1], Ching-Hsuan Tung[2] and Youli Zu[1,*]

[1]Department of Pathology and Genomic Medicine, Houston Methodist Hospital, Cancer Pathology Research Laboratory, Houston Methodist Research Institute, Houston TX 77030, USA;
[2]Molecular Imaging Innovations Institute, Department of Radiology, Weill Cornell Medical College, New York, NY 10021, USA

***** Correspondence: yzu@HoustonMethodist.org; Tel.: +1 (713) 441-4460

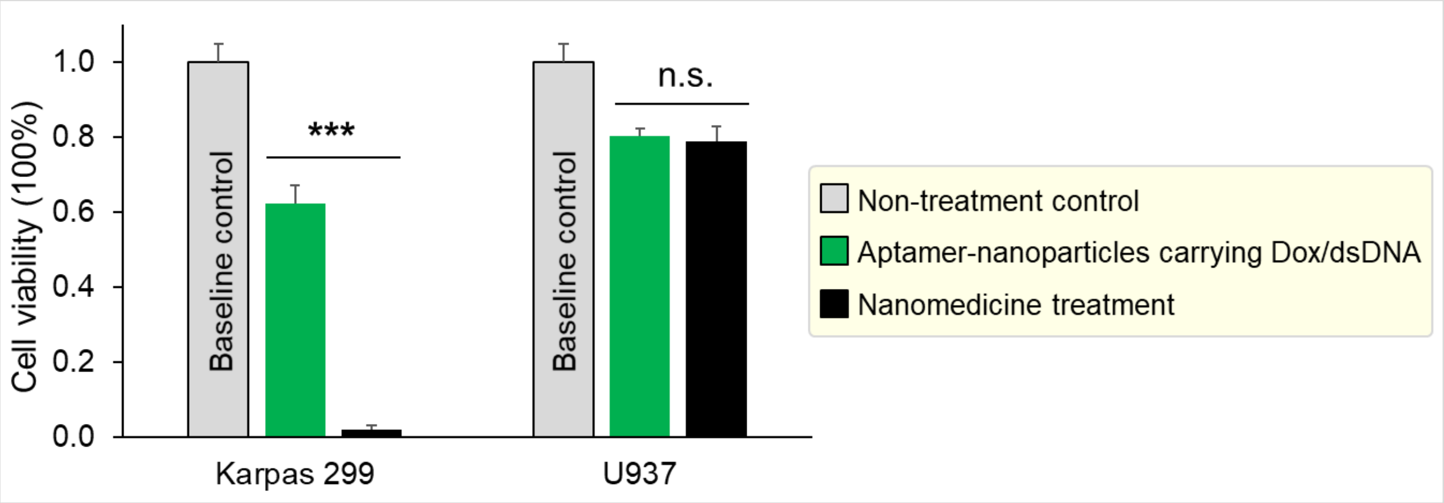

Supplemental Figure S1. Therapeutic effects of target chemotherapy alone vs. combined chemotherapy and gene therapy. Lymphoma cells were treated with the same amounts of aptamer-nanoparticles carrying Dox/dsDNA complex (no siRNA, for target chemotherapy alone) or protamine nanomedicine (1µg/ml) for combined chemotherapy and gene therapy, or vehicle alone for baseline control. Cells were harvested three days post treatment, and cell viability was evaluated as described in Figure 7. In comparing to targeted chemotherapy alone, protamine nanomedicine-mediated combination therapy achieved significant higher efficacy to treat Karpas 299 cells (*$p<0.001$*). In contrast, both aptamer-nanoparticles carrying Dox/dsDNA and the protamine nanomedicine showed the same minor effects on off target U937 cells (n.s. = not significant).
